# Novel Psychoactive Substances in Young Adults with and without Psychiatric Comorbidities

**DOI:** 10.1155/2014/815424

**Published:** 2014-07-15

**Authors:** Giovanni Martinotti, Matteo Lupi, Tiziano Acciavatti, Eduardo Cinosi, Rita Santacroce, Maria Salvina Signorelli, Laura Bandini, Giulia Lisi, Diego Quattrone, Paola Ciambrone, Andrea Aguglia, Federica Pinna, Salvatore Calò, Luigi Janiri, Massimo di Giannantonio

**Affiliations:** ^1^Department of Neuroscience and Imaging, University “G. d'Annunzio”, Chieti, Italy; ^2^Department of Clinical and Molecular Biomedicine, University of Catania, Catania, Italy; ^3^Department of Biomedical and Neuromotor Sciences DIBINEM, Bologna University, Bologna, Italy; ^4^Roma Tor Vergata University, Rome, Italy; ^5^Section of Psychiatry, Department of Neuroscience, Messina University, Messina, Italy; ^6^Catanzaro University, Catanzaro, Italy; ^7^University of Turin, Turin, Italy; ^8^Department of Public Health, Clinical and Molecular Medicine, University of Cagliari, Cagliari, Italy; ^9^SPDC, Vito Fazzi, Lecce, Italy; ^10^Institute of Psychiatry and Psychology, Catholic University Medical School, Rome, Italy

## Abstract

*Objective*. Comorbidities between psychiatric diseases and consumption of traditional substances of abuse (alcohol, cannabis, opioids, and cocaine) are common. Nevertheless, there is no data regarding the use of novel psychoactive substances (NPS) in the psychiatric population. The purpose of this multicentre survey is to investigate the consumption of a wide variety of psychoactive substances in a young psychiatric sample and in a paired sample of healthy subjects. *Methods*. A questionnaire has been administered, in different Italian cities, to 206 psychiatric patients aged 18 to 26 years and to a sample of 2615 healthy subjects matched for sex, gender, and living status. *Results*. Alcohol consumption was more frequent in the healthy young population compared to age-matched subjects suffering from mental illness (79.5% versus 70.7%; *P* < 0.003). Conversely, cocaine and NPS use was significantly more common in the psychiatric population (cocaine 8.7% versus 4.6%; *P* = 0.002) (NPS 9.8% versus 3%; *P* < 0.001). *Conclusions*. The use of novel psychoactive substances in a young psychiatric population appears to be a frequent phenomenon, probably still underestimated. Therefore, careful and constant monitoring and accurate evaluations of possible clinical effects related to their use are necessary.

## 1. Introduction

It is well known that mental disorders are accompanied by multiple comorbidities, but substance misuse is particularly common [1]. Many clinicians feel that substance misuse may be explained in some cases as a form of self-medication to improve psychopathology (depression, anhedonia, and negative symptoms) or to ameliorate the side effects of psychopharmacological treatment. Indeed, the cooccurrence between mental disorders and psychoactive substances misuse often leads to a more pernicious and difficult to treat course of illness, in terms of possible earlier age of onset, frequency and length of episodes, and diminished treatment compliance [2, 3]. Moreover, there is substantial evidence that substance misuse is a major risk factor for violence and aggression in patients with major mental disorders [4]. To get an idea of the problem dimension as a health issue, in 2010 mental and substance use disorders accounted for 183.9 million disability-adjusted life years (DALYs) or 7.4% of all DALYs worldwide. Thus, considered together, mental and substance use disorders were the leading cause of years lived with disability (YLDs) worldwide [5]. The long-term consequences of increased reactivity (sensitisation) to episodes and substances misuse and their cross-sensitisation to each other may have a number of important implications for clinical therapeutics [2, 6]. The potential cross-sensitisation among stressors, episodes, and substances misuse raises the spectre of an adverse positive feedback mechanism in each domain of illness vulnerability, with recurrences of each not only increasing responsivity to itself, but also increasing responsivity to the others [2].

Recently, beyond “classic” substances of abuse, it seems that novel (new) psychoactive substances (NPS) are determining a further sanitary issue of growing importance, especially in relation to the fast-moving and potentially unlimited nature of their online market [7]. The term “novel psychoactive substances” (NPS) has been legally defined by European Union as a new narcotic or psychotropic drug, in pure form or in a preparation, that is not scheduled under the Single Convention on Narcotic Drugs of 1961 or the Convention on Psychotropic Substances of 1971, but may pose a public health threat comparable to that posed by substances listed in those conventions (Council of the European Union decision 2005/387/JHA) [8]. NPS are often almost unknown to health professionals, mainly due to the lack of evidence-based sources of information [9]. Since 1997, more than 200 novel psychoactive compounds have been reported; out of these, 41 were reported in 2010, 60 in 2011, and 57 in 2012 [7]. These substances are most often synthesized in underground laboratories, simply modifying the molecular structure of controlled drugs, hence raising further concerns in terms of the presence of contaminating agents [8]. The World Wide Web has emerged as a primary source of information about drugs in general and NPS in particular. Drug users can obtain information through online forums, chat rooms, and blogs and find out about new products. They can also communicate with other users about their experiences, the effects of the substances, and the recommended sources and routes of delivery. This may be an issue of concern if one considers that an estimated 61% of young European people aged between 15 and 24 years typically quote the Internet as a potential source of information on drugs [10]. The number of online shops offering to supply with NPS customers residing in European countries increased from 170 in January 2010 to 314 in January 2011 and 693 in January 2012 [7]. The possibility of purchasing NPS from web sites makes these drugs very easily available to vulnerable individuals, including children and adolescents. Vulnerable consumers are targeted by aggressive marketing strategies (attractive names, colourful packaging, and free samples to test); NPS appear to be mostly unregulated, and this may facilitate their popularity as well as the users' perception of risks associated with consumption. The idea that legality can equate with safety still remains well grounded amongst some recreational users [9].

Thus, the focus on novel psychoactive substances, peculiarly in youths, has become a diffuse topic of discussion in scientific literature, underlining a growing interest for this widespread phenomenon. However, still few epidemiological data about NPS diffusion exists. Recent data by European Monitoring Centre on Drugs and Drug Addiction (EMCDDA) highlights that small percentages of tested youths have experienced NPS (around 5%), most of them obtaining drugs from friends or at parties, rather than online [11]. A UK-based research found out that almost one-third of a sample of students (446) had tried NPS at least once in their life. A Polish epidemiological study on 14511 secondary school pupils and university students has registered NPS use rate of 4.49% and 1.83%, respectively [12]. The psychoactive substances abuse issue has been recently emphasized by researchers from all over the world: Madruga et al. have gathered information on a sample of 761 Brazilians aged from 14 to 19 years old; more than half of interviewed adolescents are regular alcohol users and one out of ten is an abuser and/or dependent, while nearly 3% has used an illicit substance in the twelve months before questionnaire administration [13]. Famuyiwa and colleagues supply epidemiologic data on 4286 school pupils (mean age 15.2 years) from Lagos, Nigeria, finding that 61.8% of respondents have used one or more psychoactive substances in their lifetime [14]. Currie reports results on* Salvia divinorum* consumption from Canada's Youth Smoking Survey (sample of 42179 Canadian adolescents aged 12–17 years), evidencing that 6.2% of the subjects has used the substance at least once in their life [15]. Kelly et al. have performed a field-based survey of 1740 patrons at nightlife venues in New York City. Within the sample, 8.2% reported use of synthetic cannabinoids and 1.1% reported use of mephedrone; the findings suggest that the use of synthetic cannabinoids and mephedrone among US nightlife scenes may remain relatively low in comparison with European nightlife scenes [16]. A recent study by Bruno et al. among 693 regular ecstasy users (REU) in Australia has evidenced that more than one quarter (28%) of REU have used a new psychoactive substance in the past six months, most commonly from the stimulant class (20%, typically mephedrone 17%) rather than from the psychedelic class (13%) [17]. These behaviours and patterns of use are encouraged by the increasing phenomenon of binge drinking, widely diffused in Europe and North America [18–20].

To the best of our knowledge, no current data exist about the use of NPS among psychiatric patients. This is the first study aiming to assess both the presence and the nature of NPS misuse in a population of Italian young adults in comparison with a psychiatry patient sample.

## 2. Materials and Methods

A questionnaire has been administered to a sample of 2615 healthy subjects, aged between 18 and 26 years. The instrument has been designed by comparing different theories and points of view about abuse and addiction. The data were collected between September 2013 and January 2014; the questionnaire was self-administered in an anonymous way by our team of psychologists and psychiatrists, with the support of a peer-working group. We investigated socioeconomic characteristics (age, gender, residence, job status, level of education, and living status), alcohol use, and substance use (tobacco, caffeine, cannabis, and cocaine) with a peculiar focus on Novel Psychoactive Substances (NPS). The NPS we investigated are as follows: synthetic cannabinoids (spices), mephedrone (bath salts), methamphetamine (ice-shaboo-crystal meth), Ayahuasca, phenethylamines (Nbome-Fly-Solaris),* Salvia divinorum*, Kratom, gamma hydroxybutyric acid (GHB), methoxetamine (Special M), and desomorphine (krokodil).

The selected sample resided in different Italian cities, located in the north, centre, and south of the country, to ensure the inclusion of youths from diverse social and provenance contexts.

The same survey has been administered to a sample of 206 psychiatric patients, with DSM-5 fixed diagnoses at the time of test, excluding those with a substance use disorder. The patients were recruited in eight departments of mental health in various Italian cities, located in the north, centre, and south of the country to ensure a comparable sample. Healthy subjects were selected homogeneously by age, gender, and housing condition, following as a randomizing procedure the Snowball sampling [21]. The psychiatric subjects sample was composed of all the new inpatients admitted in the 8 recruiting centres in the period between December 2013 and March 2013.

Data collection was carried out in an anonymous and confidential way; all participants received a detailed explanation of the design of the study and a written informed consent was systematically obtained from every subject, according to the Declaration of Helsinki.

Baseline data were analysed using descriptive statistics, including means and standard deviations and frequencies and percentages. The chi-square (*χ* ^2^) test, Fisher's exact test, and nonparametric Wilcoxon-Mann-Whitney test were used for comparison of qualitative data. Quantitative variables were summarized by means and medians and compared using the Student's *t*-test. Factors with a *P* value lower than 0.25 were included in the multivariate analysis and *P* value lower than 0.05 was considered to be significant. SPSS version 14.0 was used for all analyses.

## 3. Results

The sample of 2615 healthy subjects consisted of 44.6% of males and of 55.4% of females, with a mean age of 22.01 ± 2.6 years. The sociodemographic data indicated that the majority of respondents had attended high school (66.6%), was living with parents (67.4%), and was a student (59%).

On the other hand, the sample of 206 psychiatric patients (20.9% diagnosed with schizophrenia or other psychotic disorders, 15.5% with depressive disorders, 13.1% with bipolar disorder, 27.2% with anxiety disorders, 17% with personality disorders, and 6.3% with Obsessive Compulsive Disorder) was composed of 45.6% males and 54.4% of females, with a mean age of 22.4 ± 2.7 years. 63.8% of patients had attended high school, 75.5% was living with parents, and 37.4% was a student at the time of testing (Table 1).

Habitual consumption of alcoholic beverages was significantly more common in healthy subjects than in patients (79.5% versus 70.7%; *P* < 0.003), as well as Binge Drinking behaviours (59.7% versus 47.6%; *P* < 0.001).

With regard to the association of alcohol consumption with use of other substances (37.8% in healthy subjects and 45.3% in patients), there was no statistically significant difference between two groups.

The data on the consumption of drugs indicated that the difference in cannabis use between the two groups (25.6% in healthy subjects and 29.3% in patients) was not statistically significant, while both cocaine (8.7% versus 4.6%; *P* = 0.002) and NPS use (9.8% versus 3%; *P* < 0.001) prevailed among patients.

**Figure 1:**
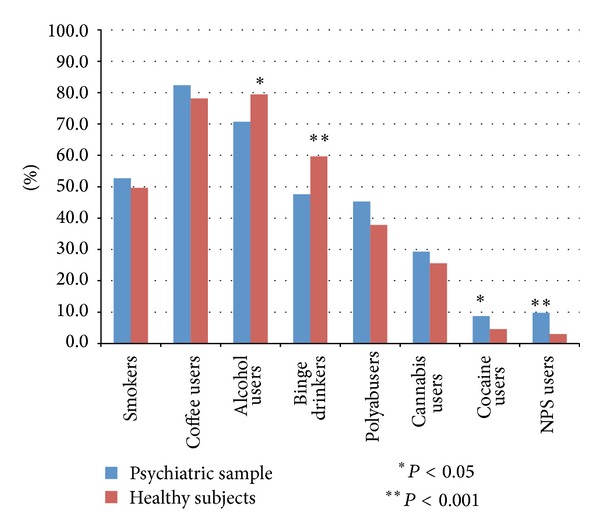
Psychoactive substances.

The differences in coffee consumption (80.4% in healthy subjects and 82.4% in patients) and cigarettes smoking (46.7% in healthy subjects and 52.7% in patients) were not statistically significant between the two groups (Figure 1).

**Figure 2:**
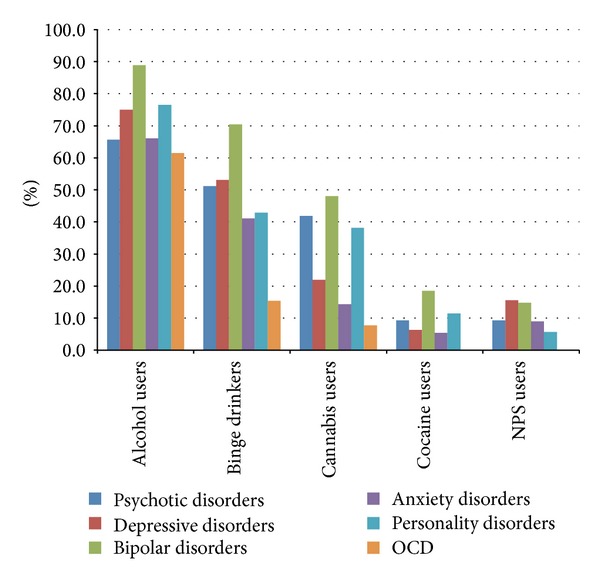
Psychoactive substances use in psychiatric patients.

Regarding the consumption of substances in the sample of psychiatric patients, those with a diagnosis of schizophrenia and other psychotic disorders used to consume alcohol in 65.6% of cases, had binge drinking behaviours in 51.2% of cases, used cannabinoids in 41.9% of cases, consumed cocaine in 9.3% of cases, and used NPS in 9.3% of cases; those diagnosed with depressive disorders consumed alcohol in 75% of cases, with a binge prevalence of 53.1%; cannabinoids were used in 21.9% of cases, cocaine in 6.3%, and NPS in 15.6%. Patient with a diagnosis of bipolar disorder consumed alcohol in 88.9% of cases, with concomitant binge drinking in 70.4% of the sample; use of cannabinoids was evidenced in 48.1% of cases, cocaine in 18.5%, and NPS in 14.8%. Those diagnosed with anxiety disorders consumed alcohol in 66.1% of cases, with a prevalence of binge behaviours of 41.1%; use of cannabinoids was reported in 14.3% of cases, cocaine in 5.4%, and NPS in 8.9%. Patients diagnosed with personality disorder drank alcohol in 76.5% of cases, had binge drinking behaviours in 42.9% of cases, and used cannabinoids in 38.2% of cases, cocaine in 11.4%, and NPS in 5.7%. Those diagnosed with DOC consumed alcohol in 61.5% of cases, with a binge prevalence of 15.4%; they smoked cannabinoids in 7.7% of cases but did not use cocaine or NPS (Figure 2).

More specifically, with regard to the use of NPS, our data revealed that among healthy subjects 1.6% had experimented with methamphetamine, 1.1% with* Salvia divinorum*, 1% with synthetic cannabinoids, 0.6% with phenethylamines, 0.3% with mephedrone, 0.3% with GHB, 0.2% with Ayahuasca, 0.2% with methoxetamine, 0.2% with desomorphine, and 0.1% with kratom.

In the psychiatric patients' sample, we evidenced that 5.8% used Synthetic Cannabinoids, 3.4% methamphetamine, 1.9% GHB, 1%* Salvia divinorum*, 1% methoxetamine, 0.5% mephedrone, 0.5% Ayahuasca, 0.5% phenethylamines, 0% kratom, and 0% desomorphine (Table 2).

## 4. Conclusions

To the best of our knowledge, this is the first paper providing some data about NPS misuse in psychiatric patients; nevertheless, it is well known that psychiatric patients are much more likely to have a substance or alcohol abuse problem than general population [22–24].

Our results show a higher prevalence of habitual consumption of alcohol and binge drinking behaviours in healthy subjects in comparison with psychiatric patients. These data may be explained with the widespread social use of alcohol and with the concept of the “drink in the company” as a social glue, while it is known that psychiatric conditions lead to marginalization and isolation. Another reason may be related to the continuous contact of psychiatric patients with medical figures and drug therapies, which might contribute to a reduction in the consumption of alcohol [25, 26].

On the other side, the consumption of NPS and cocaine was significantly higher in the patients group than among healthy subjects. These data may have different meanings: firstly, there is the possibility that the use of these substances is itself a factor able to trigger prodromal symptoms to full development; on the other hand, subjects with psychiatric disorder may be more motivated to try compounds considered harmful and/or illegal, possibly as self-medication agents. These are of course only speculations not fully justified by our data, but may represent future hypothesis that need to be investigated. The presence of a quite high percentage of patients with a diagnosis in the area of psychotic and bipolar disorders associated with the use of cannabis is of clinical interest. From our data it is not possible to understand if cannabis use represents a predisposing factor. However, this association gives further emphasis to the debated issue of a relationship between cannabis and major psychiatric disorders [3, 27, 28]. Other data on substances consumption registered for different psychiatric diagnoses were suggestive and worthy of interest despite being limited by the smallness of the subgroups.

In our study, the use of NPS in healthy subjects and psychiatric patients is statistically in favour of the patients group (9.8% versus 3%; *P* < 0.001). The situation of NPS consumption in Europe, in response to recent developments in EU drug market, is analysed by the Eurobarometer “Youth attitudes on Drugs”. The survey asked adolescents and young adults about their experiences and attitudes towards new psychoactive substances. The sample included over 12000 youths aged 15–24, randomly selected across the 27 EU Member States. Overall, 5% of the participants reported having used NPS: Ireland (16%), Poland (9%), Latvia (8.8%) and United Kingdom (8%) were way above the mean, while Italy (0.8%), Finland (1%) and Greece (1.6%) were at the bottom of the list [29]. On the other hand, our recent Italian data showed a higher percentage of NPS consumption in healthy subjects, as well as a considerable higher percentage in psychiatric patients. This could be due to selection biases, but it is also possible that the extent of NPS consumption may be growing or it was previously underestimated.

In our sample, we have evidenced a relevant of use of synthetic cannabinoids among both patients (5.8%) and healthy controls (1%). Being one of the most known and used NPS, synthetic cannabinoids may represent a significant health issue. Moreover, Spice use is apparently gaining popularity among teenagers and young adults in the US, and a European survey found similar figures, with a 7% prevalence of lifetime use in a sample aged 15 to 18 years [30, 31]. The strong psychotogenic action of synthetic cannabinoids is supposed to be due to their higher affinity for cannabinoid receptors and to their lower concentration of cannabidiol [32, 33]. Papanti et al. recently coined the term “Spiceophrenia,” to define the peculiar psychopathological characteristics of spice-induced psychosis: according to their assumptions, it is possible to hypothesize that the use of synthetic cannabinoids may “trigger” the occurrence or the relapse of psychosis in psychosis-vulnerable individuals or in patients with a prodromal psychotic syndrome [34].

Cathinones derivatives, and especially mephedrone, have become a particularly widespread phenomenon in the UK in 2010, with a peak of 23.4% of Scottish students who had used the substance at least once in their life [35, 36]. In our sample, mephedrone was used by 0.3% of healthy subjects and 0.5% of psychiatric patients. Mephedrone core activity is mainly stimulant-like, with desired effects such as mood enhancement and alertness, but may also determine the development of hallucinogenic symptoms, anxiety, agitation, and confusion, with unpredictable consequences, especially in nonhealthy users [37].

Our results indicated other potentially alarming trends of misuse, such as phenethylamines and derivatives (0.6% in healthy controls and 0.5% in patients),* Salvia divinorum* (1.1% in healthy subjects and 1% in psychiatric sample), and even pharmaceutical products (e.g., 0.3% of healthy sample and 1.9% of patients declared to have assumed GHB for recreational purpose).

Clinicians could argue that NPS may reduce the efficacy of the treatments for psychiatric disorders, worsen symptoms, and reduce the adherence to therapeutic plans. On the other hand, health and other professionals should be rapidly and accurately informed about these new and serious trends of misuse. A questionnaire administered to professionals from the departments of addiction, psychiatry, and paediatrics and emergency room in Italy has highlighted that interviewees self-reported a poor technical knowledge of NPS. 27% of the respondents confirmed not being aware if their patients had a previous history of NPS misuse and most health professionals appeared to have concerns relating to associated medical and psychopathological risks, especially in terms of aggression/psychomotor agitation. Overall, most respondents reported the need to have better access to NPS-related reliable sources of information [38].

The use of NPS represents therefore a serious issue from both a clinical and a public health point of view. For these reasons, careful and constant monitoring, accurate evaluation of possible clinical effects related to their use, and development of prevention measures are necessary to tackle the wide escalation of NPS and to contribute in improving the quality of public health on a global level.

## Figures and Tables

**Table 1:** Sociodemographic characteristics.

Variable	Psychiatric patients (%)	Healthy subjects (%)
Age	21.4 ± 2.7 years	22.01 ± 2.6 years

Gender	Male: 45.6%	Male: 44.6%
	Female: 56.4%	Female: 55.4%

Level of educational	Primary degree: 1.5%	Primary degree: 0.2%
	Middle school: 21.7%	Middle school: 8%
	High school: 63.1%	High school: 66.6%
	University: 13.8%	University: 25.1%

Job status	Student: 37.4%	Student: 59%
	Student/worker: 8.7%	Student/worker: 10.8%
	Worker: 22.3%	Worker: 19.7%
	Unemployed: 31.6%	Unemployed: 10.5%

Living status	Parents: 75.5%	Parents: 67.4%
	Friends: 9.3%	Friends: 20.6%
	Alone: 10.3%	Alone: 8.1%
	Partner: 4.9%	Partner: 4%

Psychiatric diagnosis (DSM-5)	Psychotic Disorders: 20.9%	—
	Depressive disorders: 15.5%	—
	Bipolar disorder: 13.1%,	—
	Anxiety disorders: 27.2%	—
	Personality disorders: 17%	—
	DOC: 6.3%	—

**Table 2:** Percentage use NPS.

Types of novel psychoactive substances	Percentage use (%)	
	Healthy subjects	Patients
*“Spices”* *Synthetic cannabinoids *	1%	5.8%
*“Bath salts”* *Mephedrone *	0.3%	0.5%
*“Ice-shaboo-crystal meth”* *Methamphetamine *	1.6%	3.4%
*Ayahuasca *	0.2%	0.5%
*“Nbome-fly-solaris”* *Phenethylamines *	0.6%	0.5%
*Salvia divinorum *	1.1%	1%
*Kratom *	0.1%	0%
*“Ghb”* *Gamma hydroxybutyric acid *	0.3%	1.9%
*“Special m”* *Methoxetamine *	0.2%	1%
*“Krokodil”* *Desomorphine *	0.2%	0%
